# Supplementary material for: HIVprotI: an integrated web based platform for prediction and design of HIV proteins inhibitors
Source: J Cheminform. 2018 Mar 9;10:12. doi: 10.1186/s13321-018-0266-y (PMC5845081; doi:10.1186/s13321-018-0266-y)
Supplement: Supplementary file 2 — Additional file 2. Source code of HIVProtI web server. [file 13321_2018_266_MOESM2_ESM.zip › HIVprotI_Source-code/hivprotI/head.php]

HIVprotI Pred


# HIVprotI

  

###### HIV Protein Inhibitor Prediction

- Home
- Submit
  - IC_50_
  - Percent inhibition
- Search
- Batch
- Analog
- Help
- About
  - Algorithm
  - Datasets
    - Percent inhibition
    - IC_50_
  - Source Code
  - Team
  - Contact
